# Supplementary material for: Ecological Restoration Practices within a Semi-arid Natural Gas Field Improve Insect Abundance and Diversity during Early and Late Growing Season
Source: Animals (Basel). 2022 Dec 29;13(1):134. doi: 10.3390/ani13010134 (PMC9817726; doi:10.3390/ani13010134)
Supplement: Supplementary file 1 [file animals-13-00134-s001.zip › animals-1989946 -Figure S1-S3.pdf]

animals

Article

Ecological Restoration Practices within a Semi-arid Natural Gas Field Improve Insect Abundance and Diversity during Early and Late Growing Season

Michael F. Curran, Joshua R. Sorenson, Zoe A. Craft, Taylor M. Crow, Timothy J. Robinson and Peter D. Stahl

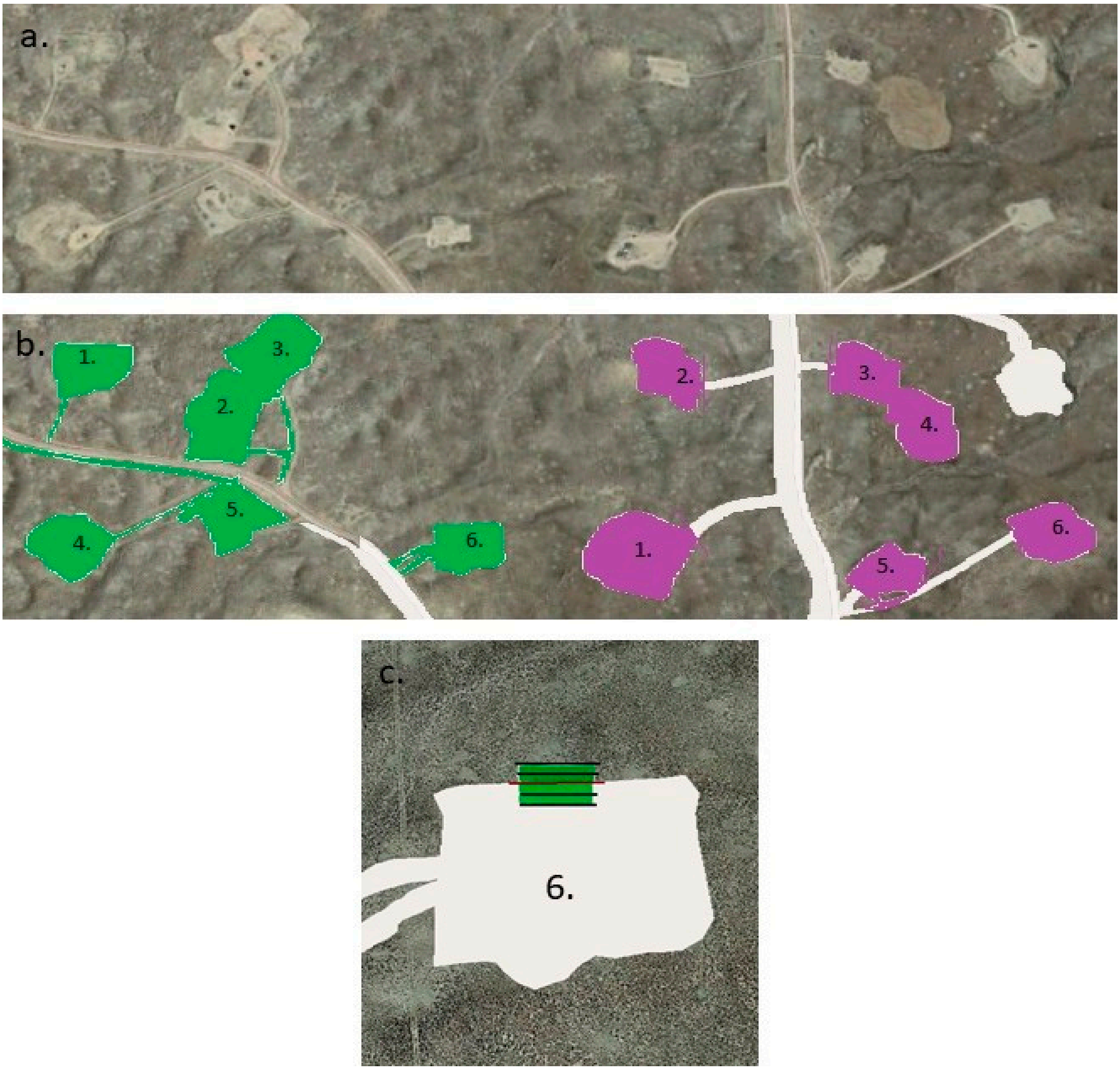

Figure S1. Overview of Study Area.

Panel a. shows an overview of the study area. Panel b. shows sites sampled in the early season colored green and sites sampled in late season colored purple. Panel c. is an example of how sweep nets and vegetation transects were run, with black lines representing 40m transect locations.

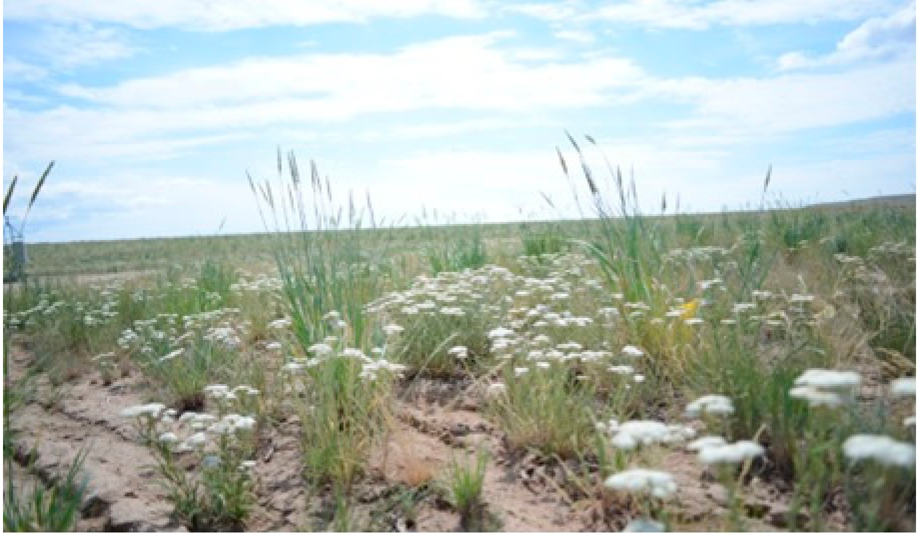

Figure S2. Vegetation on Early Season Well Pad.

An image of vegetation on reclaimed well pad sampled in early season. The white flower is yarrow.

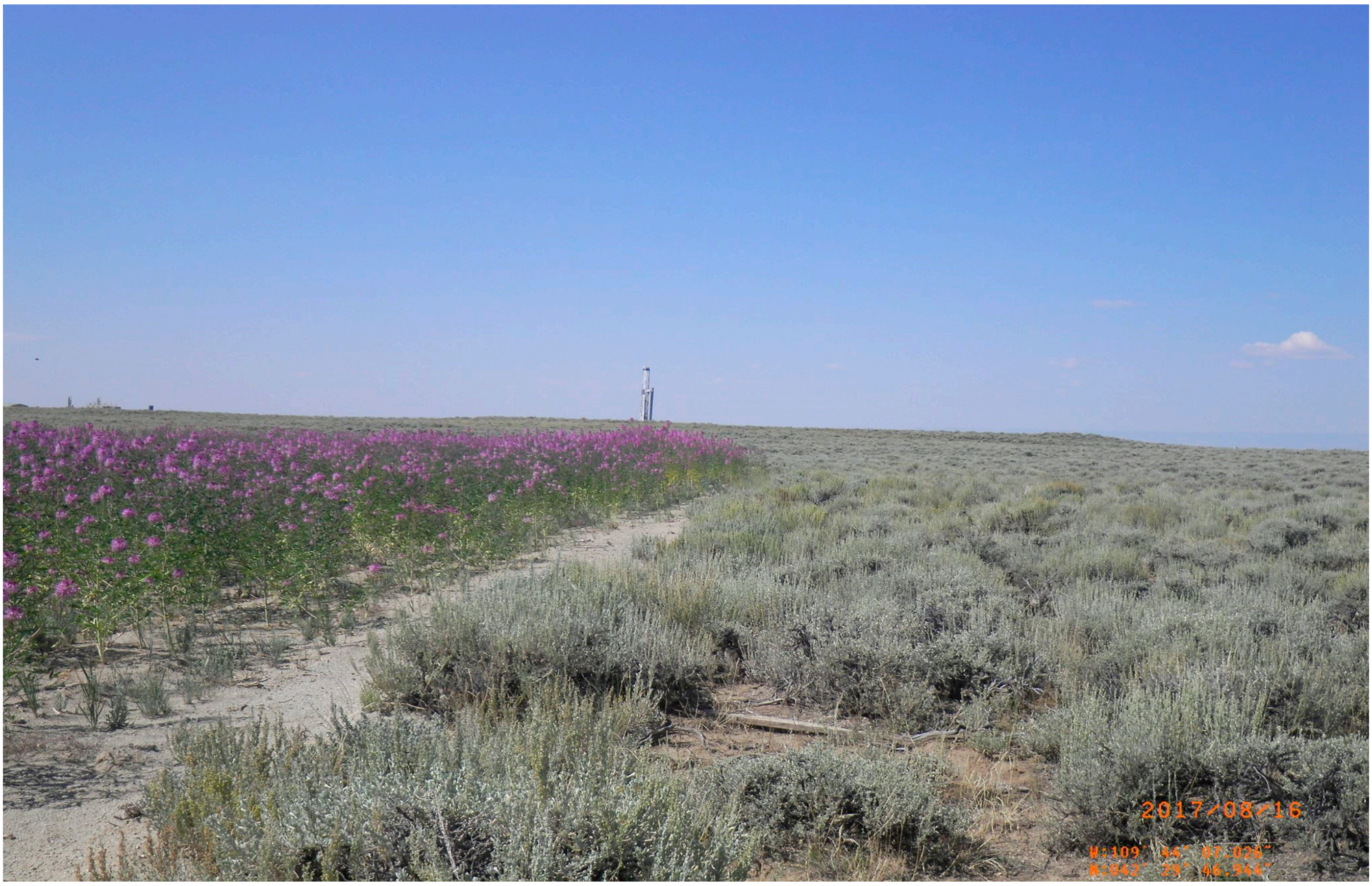

Figure S3. Vegetation on Late Season Well Pad and Reference Area.

An image at the edge of a well pad with Rocky Mountain bee plant (purple flower) abutting a reference site (sagebrush).
