# Supplementary material for: Ecological Restoration Practices within a Semi-arid Natural Gas Field Improve Insect Abundance and Diversity during Early and Late Growing Season
Source: Animals (Basel). 2022 Dec 29;13(1):134. doi: 10.3390/ani13010134 (PMC9817726; doi:10.3390/ani13010134)
Supplement: Supplementary file 1 [file animals-13-00134-s001.zip › animals-1989946 -Table S1.pdf]

animals

MDPI

Article

Ecological Restoration Practices within a Semi-arid Natural Gas Field Improve Insect Abundance and Diversity during Early and Late Growing Season

Michael F. Curran, Joshua R. Sorenson, Zoe A. Craft, Taylor M. Crow, Timothy J. Robinson and Peter D. Stahl

Table S1. Species in Seed Mix 'B1' in Jonah Field.

Achnatherum hymenoides
Elymus elymoides (Raf.) Swezey spp. *brevifolius*
Pseudoroegneria spicata (Pursh)A. Love
Leymus cinereus (Scribn. & Merr.) A. Love
Hesperostipa comata (Trin. & Rupr.) Barkworth
Atriplex canescens (Pursh) Nutt.
Sphaeralcea munroana (Douglas) Spach
Oenothera pallida Lindl.
Penstemon procerus Douglas ex Graham
Cleome serrulata Pursh
Penstemon palmeri
Artemisia tridentata spp. *Wyomingensis* Beetle & Young
Achillea millefolium L. var. *occidentalis* DC.
Eriogonum umbellatum Torr.
Artemisia tridentata Nuttall
Symphyotrichum laeve (L.) A. Love & D. Love var. *leave*
Lupinus argenteus Pursh
Linum lewisii Pursh
Krascheninnikovia lanata (Pursh) A. Meesuse & Smit
Poa secunda Presl
Erigeron engelmannii A. Nelson
